# Learning curve for minimally invasive oesophagectomy of oesophageal cancer and survival analysis

**DOI:** 10.1186/s13019-021-01712-7

**Published:** 2021-11-10

**Authors:** Yunpeng Zhao, Lei Shan, Chuanliang Peng, Bo Cong, Xiaogang Zhao

**Affiliations:** 1 Department of Thoracic Surgery, The Second Hospital of Shandong University, Jinan, 250033 China; 2 Key Laboratory of Thoracic Cancer, Shandong University, Jinan, China

**Keywords:** Oesophageal surgery, Minimally invasive surgery, Thoracoscopy, Survival analysis

## Abstract

**Purpose:**

Minimally invasive oesophagectomy is a technically demanding procedure, and the learning curve for this procedure should be explored. A survival analysis should also be performed.

**Methods:**

A total of 214 consecutive patients who underwent minimally invasive oesophagectomy were retrospectively reviewed. To evaluate the development of thoracoscopic-laparoscopic oesophagectomy and compare mature minimally invasive oesophagectomy and open oesophagectomy, we comprehensively studied the clinical and surgical parameters. The cumulative sum (CUSUM) plot was used to evaluate the learning curve for systemic lymphadenectomy. Cox proportional hazards regression analysis was performed to explore the clinical factors affecting survival.

**Results:**

The bleeding volume, operation time, and postoperative mortality within 3 months significantly decreased after 20 patients. The rise point for node dissection was visually determined to occur at patient 57 in the CUSUM plots. Patients who underwent mature thoracoscopic-laparoscopic oesophagectomy had better surgical data and short-term benefits than patients who underwent an open procedure. Cox proportional hazards regression analysis showed that the maximum diameter of the tumour cross-sectional area and the number of positive nodes significantly influenced survival.

**Conclusions:**

The results suggest that thoracoscopic-laparoscopic oesophagectomy has short-term benefits. There was no evidence that it was associated with a significantly better prognosis for patients with oesophageal cancer.

*ClinicalTrials Gov ID*: NCT04217239; January 2, 2020 retrospectively registered.

## Introduction

The incidence of oesophageal carcinoma has increased significantly over the past 20 years, and it is currently the 6th leading cause of cancer death [1, 2]. Radical surgical resection remains the primary treatment for early and locally advanced lesions [3], but it has a relatively high rate of morbidity and mortality [4], which may be attributed to lymph node dissection [5]. Minimally invasive oesophagectomy (MIE) results in low morbidity and mortality rates with equal mid- and long-term oncological outcomes [6–10].

The minimally invasive approach is performed via a distinct view of the surgical anatomy, and specialised surgical schemes and skills are required. Although learning curves and other parameters for lymph node dissection were reported over the past few years [11–14], systematic analyses of these learning curves, the clinical indexes and survival outcomes using a large number of cases or long monitoring times remain limited. From July 2010 to August 2016, 214 patients underwent MIE for oesophageal cancer and were followed up. The clinical parameters were analysed to examine the learning curve and characteristics of MIE. A clinical comparative study of mature MIE versus open oesophagectomy (OE) for oesophageal carcinoma was performed, and a Cox proportional hazards regression analysis was used to determine the clinical risk factors for overall survival.

## Materials and methods

### Patients

Between July 2010 and August 2016, 214 patients underwent MIE for oesophageal squamous cell carcinoma in the Thoracic Department, The Second Hospital of Shandong University in our cohort study. Eight of these 214 patients were converted to thoracotomy or laparotomy, 1 patient was unable to tolerate single-lung ventilation due to a history of left upper lobectomy, and the other 7 patients were converted due to bleeding control. Among the 214 patients enrolled, there were 182 males and 32 females in the MIE group. A total of 170 patients underwent oesophagectomy via open thoracotomy from August 2014 to August 2016, and these patients were defined as the open group. Data from the patients in the open group were compared to the patients who underwent thoracoscopic-laparoscopic oesophagectomy during the same clinical period. All patients were preoperatively diagnosed with oesophageal cancer by endoscopy and biopsy, and routine thoracic and abdominal enhanced computed tomography (CT) scans and endoscopic ultrasonography were used to evaluate the clinical TNM stage. A single surgical team performed all of the surgeries. The ethics committee and Medical Administration Division of the Second Hospital of Shandong University approved this study. Written informed consent was obtained from each of the enrolled patients. All methods performed in our study were performed in accordance with the relevant guidelines and regulations.

### Surgical techniques

All patients underwent curative thoracic oesophagectomy and lymphadenectomy. The MIE group underwent surgery using thoracoscopic and laparoscopic approaches. We performed cervical anastomosis (McKeown oesophagectomy) and thoracic anastomosis (Ivor-Lewis oesophagectomy). The incised margin was at least 5 cm from the superior border of the tumour.

The development process for the MIE group was divided into four stages: the first stage was from July 2010 to April 2011 and included 20 patients with relatively high mortality. The second stage was from June 2011 to July 2014 and included 37 patients. The third stage was from August 2014 to May 2015 and included 50 patients who underwent the relatively mature procedure. The fourth stage was from June 2015 to August 2016 and included 117 patients. Because this study was a retrospective study, the staging was actually performed according to natural development, and there was a pause between the stages for summarisation and improvement, especially the initial 3 stages. The results are reported consistent with the STROCSS criteria [15].

### Statistical analysis

The bleeding volume, operation time, and number of lymph nodes dissected were analysed, and these values are presented as the means ± SD. *P* values were calculated using one-way analysis of variance (ANOVA) with tests for equal variances. If heterogeneity of variance existed, one-way ANOVA was used after the data were ranked using a nonparametric test. The CUSUM plot was used to study the learning curve for systemic lymphadenectomy. The survival rate was calculated, and survival curves were drawn according to the Kaplan–Meier method.

T-tests were used to compare the bleeding volume, operation time, number of lymph nodes dissected and number of lymph node dissection sites between the MIE group and the open group. The Pearson chi-squared test was used for all of the theoretical frequencies T ≥ 5. The chi-squared test or Fisher’s exact test was used to calibrate the 4-grid data when 1 ≤ T < 5, and Fisher’s exact test was used when T < 1. The Kaplan–Meier method was used to draw the survival curve. The clinical factors were evaluated for their impact on survival using Cox proportional hazards regression analysis.

*P* < 0.05 indicated statistical significance. All analyses were performed using Stata 12.0 (StataCorp LP, College Station, TX, USA).

## Results

### Clinical data of the MIE group

The clinical and pathological features of these 214 patients are shown in Table 1. A minority of the patients received neoadjuvant chemoradiation or neoadjuvant chemotherapy, which was based on the patients’ informed consent status or economic conditions. Four patients died within 3 months due to severe complications. Postoperative adjuvant therapy was performed according to the T and N stages.

**Table 1:** The clinical, pathological and surgical features of patients in the MIE group

	1st stage	2nd stage	3rd stage	4th stage	*P* value
Cases	20	37	50	107	
Proportion of males (%)	95	89	88	81	0.340
Age mean (SD)	58.2(10.6)	63.1(10.8)	60.9(8.9)	64.7(10.4)	0.351
Proportion of smoking (%)	83	87	80	80	0.637
Neoadjuvant therapy (%)	15	16.2	22	13	0.479
*Histology type*					
Squamous Cell Carcinoma	20	37	49	106	
Adenocarcinoma	0	0	1	0	
Small Cell Carcinoma	0	0	0	1	0.543
*Differentiation grade*					
Middle or high differentiated	16	16	17	42	
Poorly differentiated	4	21	33	65	0.004
*Pathological stage*					
I	0	0	0	6	
II	13	23	28	52	
III	7	14	22	49	0.103
Negative margin	100%	100%	100%	98.13%	
Cases	20	37	50	107	
Bleeding volume (ml)	265.5 ± 107.2	199.4 ± 102.8	189.1 ± 90.7	185.9 ± 86.1	0.0092
Operation time (mins)	420.2 ± 64.3	263.5 ± 47.6	244.9 ± 60.2	248.1 ± 51.2	0.0000
Complication rate (%)	50.0	29.7	26.1	23.5	0.105
Cervical anastomotic fistula (%)	16.7(3/18)	14.8(4/27)	8.7(3/34)	9.8(6/61)	0.759
Chest anastomotic fistula (%)	0	0	0	4.3(2/46)	0.632
laryngeal nerve injury (%)	5	5.4	4	3.7	0.813
Mortality within 3 months (%)	10.0	5.4	0	0	0.0024
Lymph node dissection	12.65 ± 4.13	15.91 ± 3.36	20.16 ± 7.71	22.67 ± 7.39	0.0000
Node dissection ≥ 12	14	36	50	101	0.0000
Node dissection ≥ 18	1	12	29	71	0.0000
*Location*					
Upper or mid to upper	1	8	9	15	
Mid or lower to mid	18	23	30	65	
Lower	1	6	11	27	0.367
*Procedure*					
Ivor-Lewis	2	10	16	46	
McKeown	18	27	34	61	0.021

### Surgical characteristics of the MIE group

These data are also shown in Table 1. There was a significant difference in the bleeding volume between the 4 stages (*P *= 0.0092). There was a statistically significant difference between the 1st and 2nd stages (*P *= 0.0263), and no significant difference was found between the latter 3 stages (*P *= 0.4625). These findings were similar for the operation time, with a *P* value of 0.0000. The *P* value between the 1st stage and the 2nd stage was 0.0000, and the *P* value between the latter 3 stages was 0.1107. The subgroup analysis revealed no significant differences in the complication rates between the 4 stages (*P *= 0.105) or in the incidence of cervical anastomotic fistula, chest anastomotic fistula, and recurrent laryngeal nerve injury (*P* values of 0.759, 0.632, and 0.813, respectively). The postoperative mortality within 3 months differed significantly between the 4 stages (*P *= 0.0092). The postoperative mortality was not significantly different between the 1st and 2nd stages (*P *= 0.607), but this parameter was different between the 1st, 2nd and 3rd stages (*P *= 0.005).

**Fig. 1:**
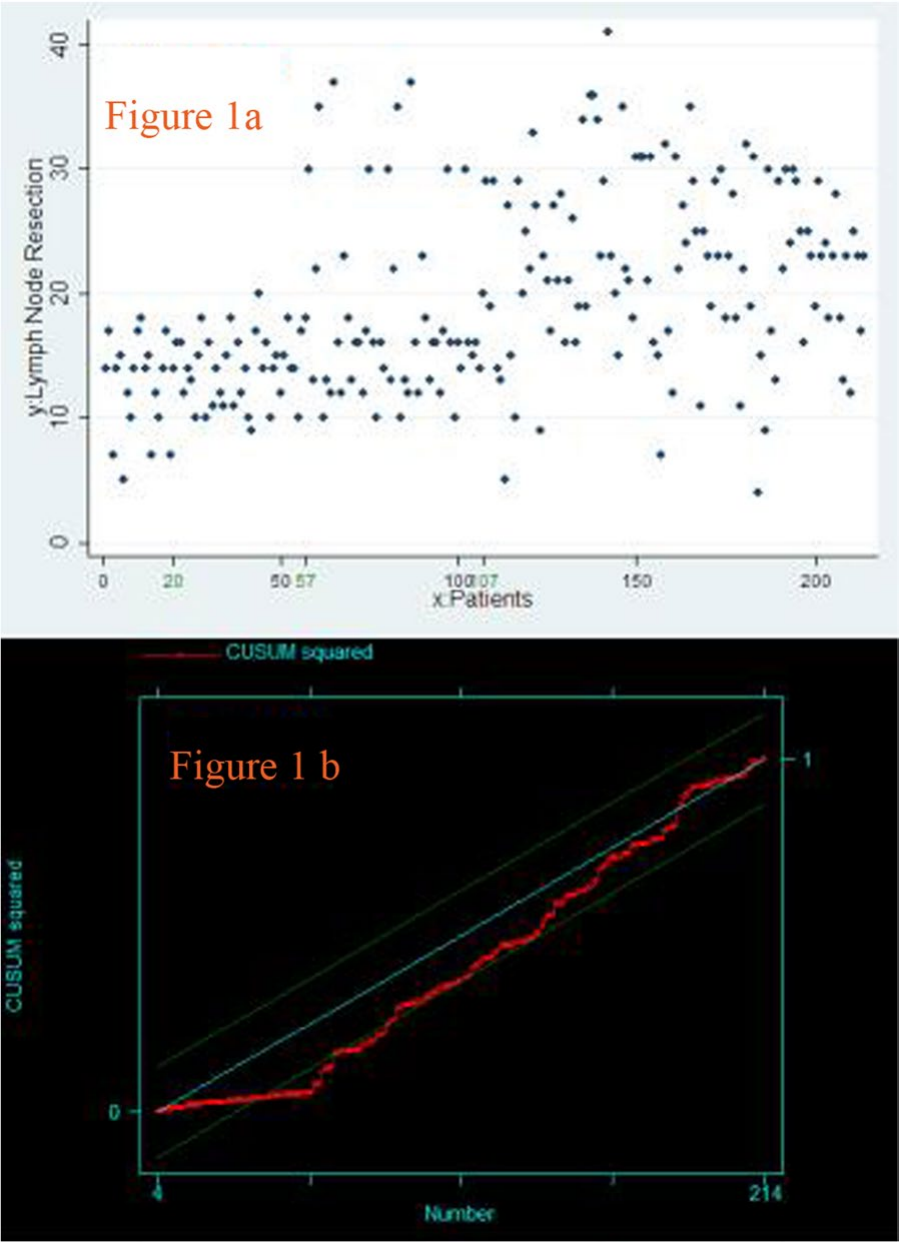
Lymph node dissection. **a** Scatter diagram of the lymphadenectomy procedure. **b** CUSUM plots of the lymphadenectomy procedure

The lymphadenectomy parameters were significantly different between the 4 stages (*P* = 0.0000) (Fig. 1a). The rise point for the number of lymph nodes dissected was visually assessed to occur at the 57th surgery (Fig. 1b), which means that the number of lymph nodes dissected increased from the 3rd stage. A comparison of stage 1 and stage 2 yielded a *P* value of 0.677, and a comparison of stage 3 and stage 4 yielded a *P* value of 0.0523. For stage 1 combined with stage 2 and stage 3 combined with stage 4, the numbers of lymph nodes dissected were 13.51 ± 3.25 and 21.26 ± 7.72 (*P* = 0.0000), respectively.

There were no significant differences in the tumour location among the 4 stages (*P* = 0.367). However, a statistically significant difference was found in the proportion of Ivor-Lewis procedures performed (*P* = 0.021). Specifically, the proportion of Ivor-Lewis procedures in the 3rd stage did not differ significantly from the former 2 stages (*P* = 0.271). However, the proportion of Ivor-Lewis procedures in the 4th stage was significantly greater (*P* = 0.010).

### Survival analysis of the MIE group

**Fig. 2:**
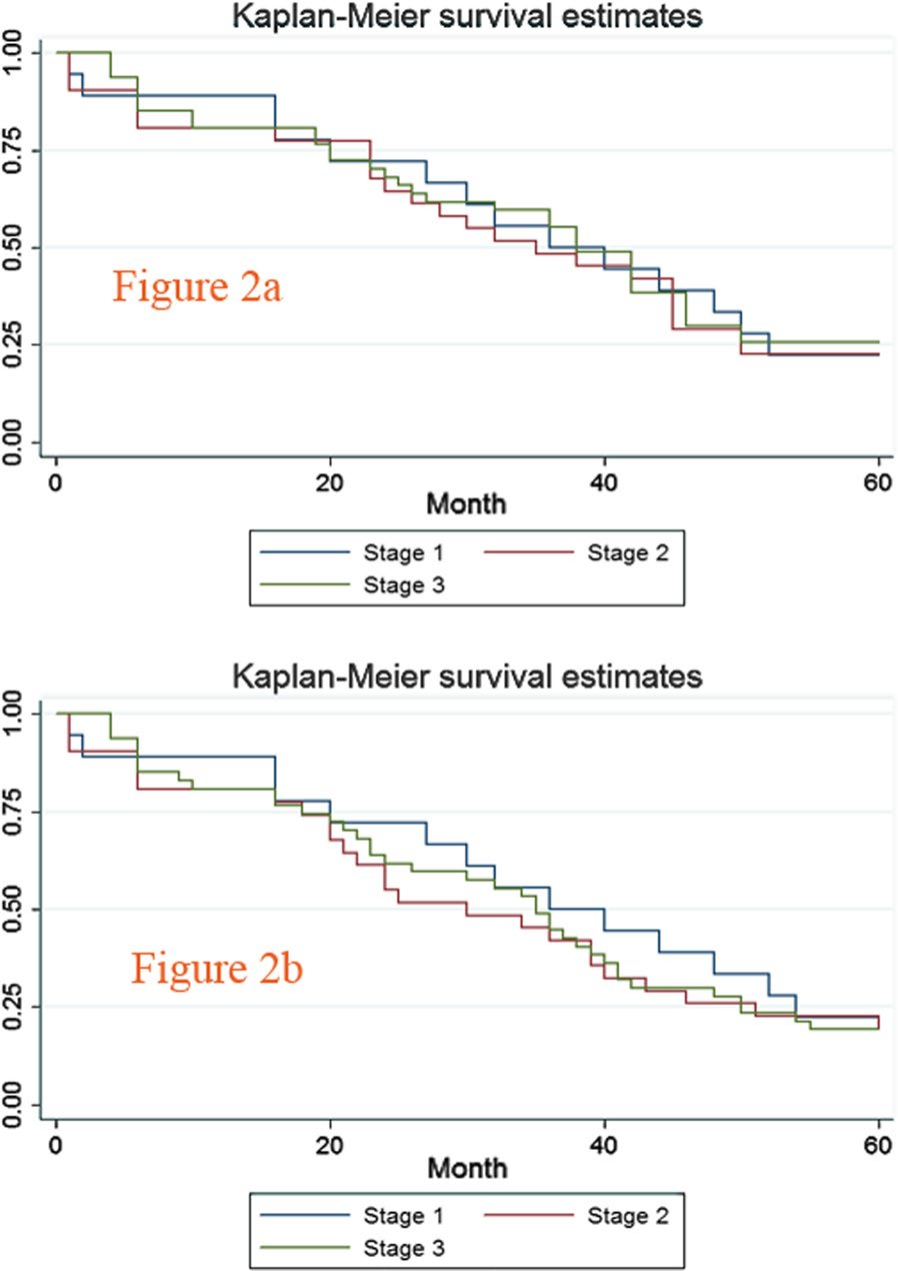
Five-year overall survival curve and disease-free survival curve of the 1st, 2nd, and 3rd stages. **a** Five-year overall survival curve of the former 3 stages. **b** Five-year disease-free survival curve of the former 3 stages

The 5-year overall survival curve and disease-free survival curve of the 1st 3 stages are shown in Fig. 2a, b. There were no significant differences in the 5-year overall survival or disease-free survival among the 3 stages (22.22%, 22.58%, and 25.53%, respectively, *P* = 0.9359) (21.22%, 19.35%, and 19.15%, respectively, *P* = 0.8361).

### Comparison between the MIE group and the open group

As seen in the above analysis, the number of lymph nodes dissected increased from the 3rd stage, which indicated that the oncological effect of MIE reached a relatively high level in the 3rd stage with a low complication rate and mortality rate. The 3rd and 4th stages were regarded as mature MIE procedures. A total of 157 patients in the 3rd and 4th stages who underwent the MIE procedure and 170 patients who underwent the open procedure were enrolled. All of the patient characteristics are shown in Table 2.

**Table 2:** Characteristics of the MIE and open groups

	MIE group	Open group	P value
Cases	157	170	
Proportion of males (%)	83	82	0.794
Age mean (SD)	63.4 (9.2)	62.0 (7.6)	0.933
Proportion of smokers (%)	79	76	0.503
Neoadjuvant therapy (%)	16	10	0.110
*Histological type*			
Squamous Cell Carcinoma	155	163	
Adenocarcinoma	1	5	
Small cell carcinoma	1	2	0.638
Differentiation grade			
Moderate to well-differentiated	59	63	
Poorly differentiated	98	107	0.922
*Pathological stage*			
I	6	18	
II	80	82	
III	71	70	0.063
Negative margin	98.73%	100%	0.230
Bleeding volume (ml)	187.2 ± 88.6	241.8 ± 127.4	0.0000
Operation time (mins)	246.1 ± 53.9	262.7 ± 65.4	0.0132
Complication rate (%)	24.2	40	0.359
Cervical anastomotic fistula (%)	9.5 (9/95)	5.5 (2/36)	0.726
Chest anastomotic fistula (%)	3.2 (2/62)	0.75 (1/134)	0.236
laryngeal nerve injury (%)	3.8	2.4	0.527
pulmonary inflammation	12.1 (19/157)	29.4(50/170)	0.0000
Mortality within 3 months (%)	0	0	
Lymph nodes dissection	21.26 ± 7.72	23.99 ± 10.15	0.0069
Node station	4.87 ± 1.43	4.16 ± 1.26	0.0000
No.106rec (right or left) (%)	78.4 (123/157)	23.5 (40/170)	0.0000
No.106rec (right and left) (%)	64.7 (102/157)	7.1 (12/170)	0.0000
No.106tbL node dissection (%)	31.8(50/157)	16.5(28/170)	0.001
Left gastric artery lymph nodes (%)	12.1 (19/157)	47.1 (80/170)	0.0000
No.107 node dissection (%)	80.4 (126/157)	80.0 (136/170)	0.937
*Paraesophageal lymph nodes (including No.105, No.108, No.110)*			
Dissection (%)	90.4 (142/157)	92.9 (158/170)	0.413
*Paracardia lymph nodes*			
Dissection (%)	41.4 (65/157)	42.4 (72/170)	0.862
*Paragastric lymph nodes*			
Dissection (%)	47.1 (74/157)	47.1 (80/170)	0.278

There was a significant difference in the bleeding volume between the MIE group and the open group (187.2 ± 88.6 ml vs. 241.8 ± 127.4 ml, *P* = 0.0000). There was a statistically significant difference in the operation time between the MIE group and the open group (246.1 ± 53.9 min vs. 262.7 ± 65.4 min, *P* = 0.0132). The difference in the incidence of postoperative pulmonary inflammation between the MIE group and the open group yielded a *P* value of 0.0000 (24.2% vs. 40%). There were statistically significant differences in the lymph node dissection parameters between the MIE group and the open group (number of lymph nodes dissected: 21.26 ± 7.72 vs. 23.99 ± 10.15, *P* = 0.0069 and number of dissection sites: 4.87 ± 1.43 vs. 4.16 ± 1.26, *P* = 0.0000). For dissecting the No. 106rec and No. 106tbL lymph nodes, the MIE group had a statistical advantage. However, the open group had a statistical advantage for dissecting the left gastric artery lymph nodes.

### Survival analysis and Cox proportional hazards regression analysis

**Fig. 3:**
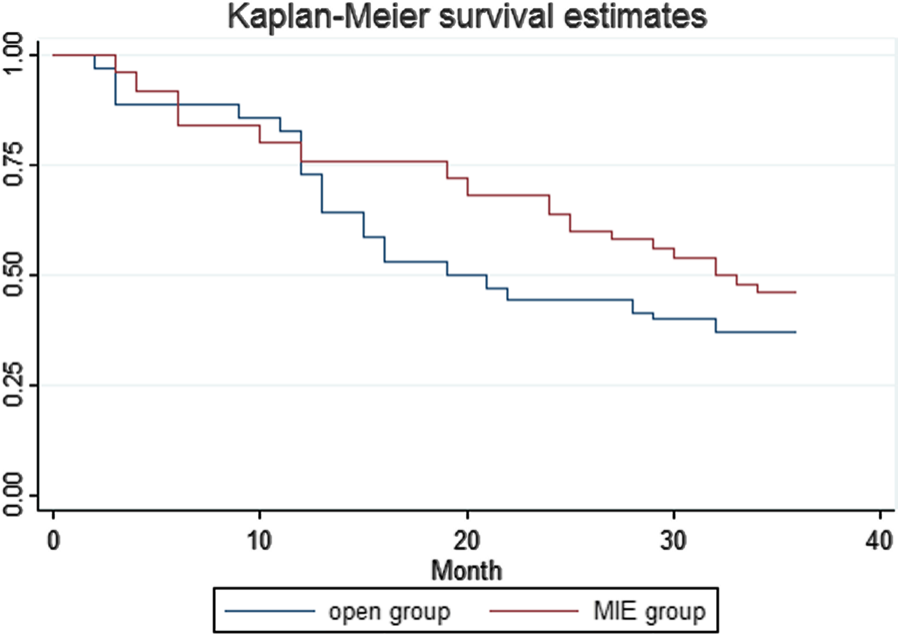
Three-year overall survival curve of the MIE group and the open group

A total of 157 patients in the 3rd and 4th stages who underwent the MIE procedure and 170 patients who underwent the open procedure were enrolled for survival analysis. The overall survival curves are shown in Figure 3, and there was no statistically significant difference in 3-year overall survival (MIE group 46.00% vs. open group 37.14%, *P*= 0.2035). A Cox proportional hazards regression was used to evaluate the impact of the clinical factors on overall survival. The following 6 parameters were screened: age, surgical procedure (MIE or open), differentiation degree (poor or middle- high), tumour excision area (≥5 cm or <5 cm), infiltration depth (T1, T2, T3), and the number of positive lymph nodes. The tumour excision area and the number of positive lymph nodes had significant impact on overall survival (P values were 0.033 and 0.003, respectively). The Cox proportional hazards model was used, and the hazard ratios (HRs) were calculated (size: Haz. Ratio = 2.632207, *P* = 0.014; Node: Haz. Ratio = 1.286079, *P* = 0.000).

## Discussion

MIE reduces postoperative pain, drainage volume and inflammatory reactions and shortens the hospital stay. However, MIE requires surgeons to have a deeper understanding of endoscopic anatomy, surgical procedure, accidental bleeding, and lymph node dissection process, which may contribute to the quality of the surgery. MIE has been performed since July 2010 in our department. Our dataset showed that surgical process proficiency was achieved after at least 57 surgeries with adequate lymph node dissection and favourable safety outcomes.

Dhamija et al. [11] reported the learning curve for lymph node resection in MIE for oesophageal cancer and suggested that the ability to dissect lymph nodes completely during MIE was affected by the surgeons’ experience, which may accumulate over time. A significant increase in experience was achieved after the first 25 cases in their study. The number of lymph nodes was regarded as an important measurement of lymphadenectomy, and different lymphadenectomy thresholds were proposed based on various scholars’ findings. Dutkowski [16] found that the diagnostic sensitivity of lymph node metastasis increased with an increasing number of dissections. When the dissection number reached 12, the sensitivity reached at least 90%, and more lymph node dissections only increased the complication rate because the diagnostic sensitivity reached a plateau. Rize [17] believed that the ideal number of lymph node dissections should be no less than 18. There was no significant difference in the pathological characteristics between the 4 stages except for differentiation. The numbers of lymph nodes dissected in each stage were 12.65 ± 4.13, 15.91 ± 3.36, 20.16 ± 7.71, and 22.67 ± 7.39 with a significant difference between the stages. To intuitively observe the upward trend, scatter plots were drawn, and the cumulative sum was calculated. The inflection point was observed at the 57th case in the present study, which suggests that proficiency in lymphadenectomy was gained after 57 patients underwent the procedure. Oncological benefits were implied with this progress. These benefits were due to the actions implemented during the 2nd stage, including performing hybrid surgeries and attending academic conferences.

MIE is divided into the McKeown technique (cervical anastomosis) and Ivor-Lewis technique (chest anastomosis). Surgeons prefer the former technique because of the maturity of the surgical procedure [18], and the latter technique is not widely performed due to its difficulty and the risk of the occurrence of a thoracic fistula. The surgical position and anastomosis type were also different in each clinical unit [19]. However, the Ivor-Lewis method was suggested as less invasive, with a shorter operation time, lower anastomotic leakage rate and lower recurrent laryngeal nerve injury rate than the McKeown method [20]. An increase in the proportion of Ivor-Lewis procedures appeared during the 4th stage in our series, after which the lymphadenectomy proficiency reached a relatively high level.

The importance of the surgical procedure was for accurate staging and an improvement in survival. The differences in 5-year overall survival and disease-free survival between the 1st 3 stages were not significant (22.22%, 22.58%, 25.53%, *P* = 0.9359) (21.22%, 19.35%, 19.15%, *P* = 0.8361), which may be due to the standard surgery procedure and sufficient lymph node dissection, even when performed in the 1st stage (12.65 ± 4.13).

We retrospectively analysed 327 patients who underwent oesophagectomy for oesophageal cancer at our institution. A total of 157 patients underwent MIE (from the 3rd stage), and 170 patients underwent open oesophagectomy (OE). The MIE group had a smaller bleeding volume (*P* = 0.0000) and shorter operation time (*P* = 0.0132) than the OE group. There was no significant difference in the complication rates between the two groups. However, the MIE group had a lower incidence of postoperative pulmonary inflammation (12.1% vs. 29.4%, *P* = 0.0000), which is very similar to the results of the randomised controlled trial of Biere et al. [21] (12% vs. 34%, *P* = 0.005). According to Parameswaran et al. [22] and Verhage et al. [23], the postoperative respiratory complication rate of OE was higher than MIE. This result may be due to the large incision, rib fractures, respiratory muscle detachment, injury and repair of the diaphragm, and retention of sputum. Therefore, MIE was superior to OE for protecting respiratory function.

The open group had more lymph nodes dissected than the MIE group (23.99 ± 10.15 vs. 21.26 ± 7.72, *P* = 0.0069), but the number of lymph nodes dissected in the MIE group was similar to a previous study [24]. Lymph nodes were dissected from more sites in the MIE group than the OE group (4.87 ± 1.43 vs. 4.16 ± 1.26, *P* = 0.0000), which may be due to the comprehensive view available with the thoracoscope. Subgroup analysis showed that the dissection rates of the No. 106rec (right or left) lymph nodes reached 78.4% in the MIE group and 23.5% in the open group (*P* = 0.0000). The No. 106rec (both right and left) lymph node dissection rate in the MIE group was 64.7%, which was significantly different from the 7.1% in the open group (*P* = 0.0000). No.106tbL lymph node dissection followed a similar trend (31.8% vs. 16.5%, *P* = 0.001). However, the open group had a statistical advantage (47.1% vs. 12.1%, *P* = 0.0000) in left gastric artery lymph node dissection, which reflects the capability limitations of the thoracic surgeon during laparoscopic abdominal lymphadenectomy.

Survival outcomes are crucial for the treatment of carcinoma. The 1-year survival rates between MIE and OE were not significantly different [25]. Li et al. [26] reported that the 1-, 2-, and 3-year survival rates of patients who underwent MIE were not significantly different from patients who underwent the open procedure, but the proportion of early-stage disease in the MIE group was higher than open procedure group. A systematic review [27] also provided information that the proportion of early-stage disease in the MIE group exceeded the open procedure group, which may be inevitable while the surgeons are still learning. Mitzman et al. [28] performed a propensity analysis using data from the National Cancer Database of the United States, and equivalent oncological outcomes and survival outcomes were found for the MIE and open procedure groups. No significant differences in long-term survival were found in patients who underwent robotic-assisted minimally invasive oesophagectomy (RAMIE), MIE or OE. Therefore, surgeon expertise and experience may be the most important aspect [29]. The overall 3-year survival rate did not differ significantly between the groups in our series (MIE group 46.00% vs. open group 37.14%, *P* = 0.2035). This study did not provide evidence of improvements in the survival outcomes of patients who underwent MIE. Therefore, a Cox proportional hazards regression was used to evaluate the effects of clinical factors on survival for patients who underwent radical surgery with curative intent. The clinical parameters included age, surgical procedure (MIE or open), differentiation degree (poor or middle-high), tumour excision area (≥ 5 cm or < 5 cm), infiltration depth (T1, T2, T3), and the number of positive lymph nodes. The maximum diameter of the tumour excision area and the number of positive lymph nodes significantly influenced postoperative survival. There was no evidence that the surgical procedure (MIE or open) exerted significant influence on the prognosis of patients with oesophageal cancer.

There are some limitations to this study. The management of chest drainage and nasogastric drainage and inflammatory factors were not considered, but these factors are important indicators for presenting a learning curve. The number of lymph nodes was used as a reflection of lymphadenectomy completeness, and this number may be influenced by a fracturing of the nodes or pathological diligence. Our study was a retrospective analysis, and challenging cases were avoided in the early stage of the learning process. Therefore, selection bias was inevitable. Only a few patients received neoadjuvant therapy, which was recommended by the medical guidelines.

## Conclusion

Our findings provide evidence of the learning curve for MIE and its short-term benefits. However, the results provide no evidence that MIE and OE contribute differently to postoperative survival outcomes.

## Data Availability

Available on request.

## Abbreviations

OE: Oesophagectomy
CUSUM: Cumulative sum
MIE: Minimally invasive oesophagectomy
OE: Open oesophagectomy
CT: Computed tomography
ANOVA: Analysis of variance
RAMIE: Robotic-assisted minimally invasive oesophagectomy

## References

[1] Jemal A, Bray F, Center MM (2011). Global cancer statistics. CA Cancer J Clin.

[2] Tew WP, Kelsen DP, Ilson DH (2005). Targeted therapies for esophageal cancer. Oncologist.

[3] Ruurda JP, van der Sluis PC, van der Horst S (2015). Robot-assisted minimally invasive esophagectomy for esophageal cancer: a systematic review. J Surg Oncol.

[4] Raymond DP, Seder CW, Wright CD (2016). Predictors of major morbidity or mortality after resection for esophageal cancer: a society of thoracic surgeons general thoracic surgery database risk adjustment model. Ann Thorac Surg.

[5] Jamieson GG, Lamb PJ, Thompson SK (2009). The role of lymphadenectomy in esophageal cancer. Ann Surg.

[6] Osugi H, Takemura M, Higashino M (2003). A comparison of video-assisted thoracoscopic oesophagectomy and radical lymph node dissection for squamous cell cancer of the oesophagus with open operation. Br J Surg.

[7] Schoppmann SF, Prager G, Langer FB (2010). Open versus minimally invasive esophagectomy: a single-center case controlled study. Surg Endosc.

[8] Parameswaran R, Veeramootoo D, Krishnadas R (2009). Comparative experience of open and minimally invasive esophagogastric resection. World J Surg.

[9] Ben-David K, Sarosi GA, Cendan JC (2012). Decreasing morbidity and mortality in 100 consecutive minimally invasive esophagectomies. Surg Endosc.

[10] Kinjo Y, Kurita N, Nakamura F (2012). Effectiveness of combined thoracoscopic-laparoscopic esophagectomy: comparison of postoperative complications and midterm oncological outcomes in patients with esophageal cancer. Surg Endosc.

[11] Dhamija A, Rosen JE, Dhamija A (2014). Learning curve to lymph node resection in minimally invasive esophagectomy for cancer. Innovations.

[12] Xie X, Fu JH, Wang JY (2012). Analysis of learning process of video-assisted minimally invasive esophagectomy for thoracic esophageal carcinoma. Zhonghua Wei Chang Wai Ke Za Zhi.

[13] Dhamija A, Rosen JE, Dhamija A (2014). Learning curve to lymph node resection in minimally invasive esophagectomy for cancer. Innovations Phila.

[14] Zhao Y, Dong X, Cong B (2016). Development pattern on lymph node resection in minimally invasive esophagectomy and 2-year survival analysis. Thorac Cardiovasc Surg.

[15] Agha R, Abdall-Razak A, Crossley E, et al, for the STROCSS Group. The STROCSS 2019 guideline: strengthening the reporting of cohort studies in surgery. Int J Surg 2019;72:156–165. 10.1016/j.ijsu.2019.11.002 31704426

[16] Dutkowski P, Hommel G, Bottger T (2002). How many lymph nodes are needed for an accurate pN classification in esophageal cancer? Evidence for a new threshold value. Hepatogastroenterol.

[17] Rizk NP, Ishwaran H, Rice TW (2010). Optimum lymphadenectomy for esophageal cancer. Ann Surg.

[18] Liu BX, Li Y, Qin JJ (2013). Comparison of modified McKeown minimally invasive approach and the left chest-neck incision approach esophagectomy to treat cancer of mid—to—distal thoracic esophagus. Chin J Thorac Cardiovasc Surg.

[19] Ben-David K, Sarosi GA, Cendan JC (2010). Technique of minimally invasive Ivor-Lewis esophagogastrectomy with intrathoracic stapled side-to-side anastomosis. J Gastrointest Surg.

[20] Xie MR, Liu CQ, Guo MF (2014). Short- term outcomes of minimally invasive ivor-lewis esophagectomy for esophageal cancer. Ann Thorac Surg.

[21] Biere SS, vail Berge Henegouwen MI, Maas KW, et al. Minimally invasive versus open oesophagectomy for patients with oesophageal cancer: a multicentre, open-1abel, randomised controlled trial. Lancet 2012;379:1887–92. 10.1016/S0140-6736(12)60516-9 22552194

[22] Parameswaran R, Veeramootoo D, Krishnadas R (2009). Comparative experience of open and minimally invasive esophagogastric resection. World J Surg.

[23] Verhage RJ, Hazebroek EJ, Boone J (2009). Minimally invasive surgery compared to open procedures in esophagectomy for cancer: a systematic review of the literature. Minerva Chir.

[24] Luketich JD, Pennathur A, Awais O (2012). Outcomes after minimally invasive esophagectomy: review of over 1000 patients. Ann Surg.

[25] Lazzarino AI, Nagpal K, Bottle A (2010). Open versus minimally invasive esophagectomy: trends of utilization and associated outcomes in England. Arm Surg.

[26] Li J, Tan L, Wang Q (2013). A retrospective study for Ivor-Lewis and McKeown esophagectomy: mininally invasive versus open esophagectomy. Chin J Thorac Cardiovasc Surg.

[27] Dantoc MM, Cox MR, Eslick GD (2012). Does minimally invasive esophagectomy (MIE) provide for comparable oncologic outcomes to open techniques? A systematic review. J Gastreintest Surg.

[28] Mitzman B, Lutfi W, Wang CH (2017). Minimally invasive esophagectomy provides equivalent survival to open esophagectomy: an analysis of the national cancer database. Semin Thorac Cardiovasc Surg.

[29] Weksler B, Sullivan JL (2017). Survival after esophagectomy: a propensity-matched study of different surgical approaches. Ann Thorac Surg.

